# Lossless and Near-Lossless L-Infinite Compression of Depth Video Data

**DOI:** 10.3390/s25051403

**Published:** 2025-02-25

**Authors:** Mohammad Ali Tahouri, Alin Adrian Alecu, Leon Denis, Adrian Munteanu

**Affiliations:** 1 Department of Electronics and Informatics (ETRO), Vrije Universiteit Brussel, Pleinlaan 2, 1050 Brussels, Belgium; ldenis@etrovub.be; 2 Faculty of Engineering in Foreign Languages (FILS), Universitatea Nationala de Stiinta si Tehnologie Politehnica Bucuresti, Splaiul Independentei 313, 060042 Bucharest, Romania; alin_adrian.alecu@upb.ro

**Keywords:** depth map, lossless and near-lossless compression, foreground-background segmentation, L-infinite, quantization, MAXAD

## Abstract

The acquisition of depth information sensorial data is critically important in medical applications, such as the monitoring of the elderly or the extraction of human biometrics. In such applications, compressing the stream of depth video data plays an important role due to bandwidth constraints on transmission channels. This paper introduces a novel lightweight compression system that encodes the semantics of the input depth video and can operate in both lossless and L-infinite near-lossless compression modes. A quantization technique that targets the L-infinite norm for sparse distributions and a new L-infinite compression method that sets bounds on the quantization error is proposed. The proposed codec enables the control of the coding error on every pixel in the input video data, which is crucial in medical applications. Experimental results show an average improvement of 45% and 17% in lossless mode compared to standalone JPEG-LS and CALIC codecs, respectively. Furthermore, in near-lossless mode, the proposed codec achieves superior rate-distortion performance and reduced maximum error per frame compared to HEVC. Additionally, the proposed lightweight codec is designed to perform efficiently in real time when deployed on an embedded depth-camera platform.

## 1. Introduction

Presently, the importance of image and video compression is undeniable in medical and computer vision applications, where the compression of depth video data is crucial for optimizing bandwidth usage while preserving the integrity of the original scene. Effective video compression is vital in enhancing healthcare delivery, which calls for further research in order to improve the technological capabilities of subsequent tasks making use of compressed video data. Many standards have been developed during recent decades for image and video compression. The standards are designed to encode signals using the available pixel information while optimizing for maximum compression efficiency and performance.

The basic concept of image compression is to reduce the size of the original image by representing the input with the fewest possible bits. Image compression techniques can be broadly categorized into two types based on the presence of error: lossless and lossy (or near-lossless) compression [1]. In lossless compression, the original image is perfectly reconstructed at the decoder, ensuring that there is no error between the original and reconstructed images. In lossy compression, some data are shrunk or discarded to achieve a significantly higher compression ratio, resulting in a potential loss of image quality.

Several studies provide theoretical reviews of image compression techniques with a particular focus on lossless compression in medical applications [2,3], while others have concentrated on the practical part [4,5,6,7]. In practical applications, these papers mainly utilize CT grayscale or MRI images. In [8], a spatial domain image codec is introduced. This technique involves dividing the image into multiple blocks and calculating the difference between minimum and maximum pixel values within each block. Then, the minimum pixel value of the block is subtracted from each pixel, and the resulting values are stored and encoded for the new block. In [9], the same idea of spatial domain image compression is used, but the Lempel–Ziv–Welch (LZW) algorithm is applied to encode the subtraction value. Both methods are designed only for lossless compression of images. Lastly, a comprehensive review of classical and novel image compression methods based on the region-of-interest (ROI) [10] is presented in [11] that emphasizes the growing importance of image compression in various aspects of our digital life.

Depth images are data sources that encode a depth value in each pixel, representing the distance of that pixel from the depth camera. The depth map is a representation of a scene in two dimensions grayscale. These types of data sources have already found applicability in numerous domains, including the medical one, where major hospitals and medical centers world-wide commonly use cameras for patient monitoring. In this respect, various algorithms have been proposed in the literature that aim at compressing the depth map images using advanced methods. For instance, the work of Tabus et al. [12] encodes both the contours and the depth value from the depth map. Their method, referred to as CERV (crack-edge-region-value), begins from an initial representation of the image, utilizing binary vertical and horizontal edges of the region contours, along with the depth values assigned to each region. This approach is then used in [13] to introduce the Anchor Points Coding (APC) technique, where the anchor points of contours are encoded using a context tree algorithm. Schiopu and Tabus also developed their work of Greedy rate-distortion Slope Optimization (GSOm) [14] by proposing a progressive coding technique for lossy-to-lossless compression methods [15]. Furthermore, a new approach in [16] suggests the single depth intra mode to efficiently encode the smooth area within a depth image. The idea behind this is to simply reconstruct the current Coding Unit (CU) as a smooth area with a single depth sample value, thereby enhancing the encoding efficiency of that area within the depth map by incorporating a leaf merging of the pixel values. A bit depth compression by estimating the packet size based on the data sensor pattern is proposed in [17]. This algorithm performs only in lossless mode.

For video coding, many standards and algorithms have been developed. The foundational standards of video compression have evolved over the decades, beginning with the introduction of the Advanced Video Coding (AVC/H.264) standard in 2003 [18], followed by the release of the improved High-Efficiency Video Coding (HEVC/H.265) standard in 2012 [19] culminating with the completion of the last standard Versatile Video Coding (VVC/H.266) in 2021 [20]. The standards employ both inter-coding and intra-coding. In intra-coding, each frame in the video is encoded independently without reference to other frames. In contrast, in inter-coding, the frames are encoded using prediction mechanisms based on the previously decoded reference frames. Inter-coding improves compression performance compared to intra-coding at the expense of increased computational complexity. Existing survey papers show that compression performance has steadily improved over the years by adopting increasingly complex prediction and compression mechanisms [21,22,23].

Typical lossy compression systems, as cited above, employ the L2 metric to drive the compression process. However, L2 is a global metric that enables minimizing the overall coding error for a given bit rate. Essentially, the coding system minimizes the L2 distortion, corresponding to minimizing the mean square error between the original and reconstructed images for any given bit budget. However, such a global metric may be globally optimal, but it may also be subject to large local errors. In depth image and video data, the pixel values correspond to the actual depth from the scene to the camera plane. Controlling the coding error at the level of every pixel is thus of critical importance in order to prevent locally large depth errors that may affect subsequent processing tasks and medical decisions. What would be of interest in medical applications is to minimize the coding error at the level of every pixel for a given bit budget. This corresponds to the use of the L-infinite metric to drive the coding process.

This paper proposes a novel lossless and near-lossless L-infinite codec for video processing in medical applications, with a focus on elderly monitoring and fall detection. The compression module is of critical importance in reducing the size of streamed video, but in this respect, it is essential that the encoding process introduces either no errors (lossless) or minimal, controlled errors (near-lossless). The work described herein addresses L-infinite compression, thus guaranteeing a bounded distortion (error) in the reconstructed video. The proposed L-infinite codec is computationally lightweight, being implemented on an embedded platform (depth-camera sensor), demonstrating the practical applicability of the proposed system on embedded devices. The primary contributions of this work are as follows:

- A novel L-infinite codec for depth video data compression that preserves the semantic interpretation of the scene.
- An original quantizer that targets the L-infinite norm for sparse (discontinuous) residual distributions.
- A lightweight encoder optimized for real-time deployment on embedded platforms used in medical applications.

The remainder of this paper is organized as follows. Section 2 reviews the related works. Section 3 details the principal methodology in which we describe the architecture of our codec. Section 4 presents the experimental results following the deployment of the codec. Section 5 discusses the results. Finally, the conclusion is presented in Section 6.

## 2. Related Works

A lossless compression technique for high-precision depth maps based on pseudo-residuals is introduced in [24]. The entire process involves two sub-processes: preprocessing of depth maps followed by deep lossless compression of the processed maps. The proposed method is then used for the compression of depth images rather than for streaming depth video data. In [25], a real-time compression library called B^3^D is introduced for lossless and lossy compression in microscopy images. This library is built on the CUDA [26] architecture for GPU-based compression. Another approach for depth compression is described in [27], where a two-dimensional gradient feature—Corner Points (CPs)—is used to simultaneously accelerate the Coding Unit/Prediction Unit (CU/PU) size decision and intra-mode selection in 3D-HEVC. A corner point is defined as a point that has two main directions around its neighborhood and is used to detect a multi-directional edge. An experimental comparison of recent novel video compression standards is detailed in [28]. However, it is essential to note that all these algorithms do not bound the error on the decoder side, which means there is no possibility to control the localized distortion.

Depth medical image compression based on an optimized JPEG-XT [29] algorithm is introduced in [30]. This algorithm is applied to CT and MRI images for lossless and lossy compression. The algorithm in [31] is proposed for near-lossless and 3D reconstruction using depth-of-field segmentation and uses a method to extract the foreground and encode using the JPEG2000 standard [32].

Among the versions of the JPEG family, the JPEG-LS [33,34] encoder is a standard known for low computational complexity and memory requirements. Consequently, we selected JPEG-LS because it has a more straightforward implementation and lower complexity on embedded devices such as depth-camera sensors. The process begins with a predictive step where the value of each pixel is estimated based on neighboring pixels using a simple yet effective prediction model. The differences between the actual and predicted values, known as prediction residuals, are then computed. To adapt to varying image characteristics, JPEG-LS employs a context model that categorizes regions of the image based on local gradients. These residuals are encoded using Golomb-Rice codes, which are optimal for the statistical distributions of prediction residuals, ensuring efficient representation. Finally, it should be noted that JPEG-LS supports both lossless and lossy (near-lossless) encoding. However, despite the performance of these JPEG family standards for depth compression, according to our experiments, they do not achieve satisfying compression rates when they are used as a standalone solution. To improve the performance, we added a preprocessing block to our proposed codec, enhancing overall compression efficiency.

## 3. Methodology

In this section, we describe the architecture of the proposed coding system for video depth data. The proposed codec performs intra-frame coding wherein the frames are encoded independently. This design choice enables low computational complexity, deployment on lightweight embedded platforms, and L-infinite compression of each depth frame when operating in lossy compression mode. Additionally, we use information from successive frames to create a reference background. For the encoding part, we employ two inputs for each video frame, namely the depth frame itself, which is a 16-bit depth map, and an 8-bit segmentation map associated with each frame. Details are given next.

### 3.1. Encoder Architecture

**Figure 1:**
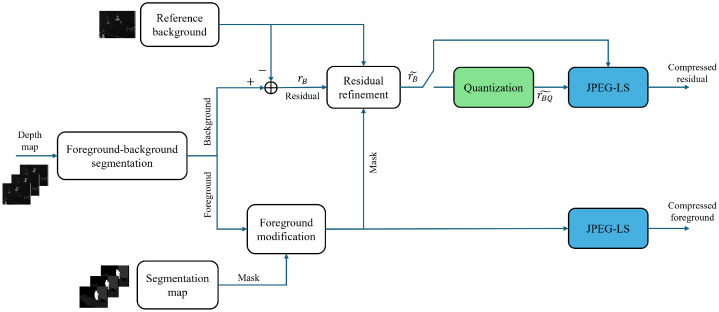
Block diagram architecture of the proposed encoder for depth video compression.

Figure 1 shows the main architecture of the encoding process. We split the foreground and background in the first stage using the segmentation map. Foreground-background segmentation is carried out using lightweight segmentation, whereby humans are semantically segmented from the background. The extracted background is then subtracted from the reference background in order to obtain a residual image. The reference background is another crucial component of the architecture that significantly impacts the compression performance. The reference background is computed based on a specific number of static frames acquired at the beginning of the encoding process. We note that whenever there is a change in the camera’s position, the reference background is updated. The movement in the camera position is detected according to changes in segmentation labels in the reference background. To create the reference background, we select the pixels that are labeled as a static object across all the stored frames, then choose the most frequent value as a depth value for the reference background. This approach ensures that the reference background accurately represents the static elements in the scene, facilitating more efficient compression. The residual image then needs to be refined according to the reference background and foreground. In the refinement block, the residual pixels labeled foreground are set to zero.

The next important block is the quantization block. It should be noted that the proposed codec supports two types of compression. For lossless compression, we bypass the quantization block, while for near-lossless compression (L-infinite compression), we apply quantization only for the residual image and losslessly encode the foreground image. The last block has the role of losslessly encoding the resulting residue data and foreground, which we achieve using JPEG-LS encoding. We detail the encoder blocks in the following sections.

#### 3.1.1. Foreground-Background Segmentation

The first block in our architecture is foreground-background segmentation, where we separate each frame into the background and foreground components. In this sense, we utilize a segmentation map, which is generated using lightweight machine-learning-based classifiers that streamline our processes to collect, store, index, and annotate data. Additionally, a feature extraction model based on multi-tracking algorithms is employed to develop lightweight and efficient classifiers. To achieve this, ResNet 3D [35], which is a well-known and widely adopted deep learning architecture, is utilized. This model is specifically designed to capture both spatial and temporal features from video data efficiently. By leveraging ResNet 3D, objects within the video frames are assigned unique tracking IDs, enabling precise annotation. Subsequently, segmentation labels are generated based on these annotated objects, ensuring an accurate and structured segmentation map representation of the video data. According to the labels in the segmentation map, we generate a mask, which we subsequently use to extract precise foreground and background information. The foreground contains only information about the moving objects in the scene.

#### 3.1.2. Quantization

The quantization process involves mapping pixel values within a specific range (or interval) to a representative value, known as the assignment point. The quantization block helps us to lower the bit rate by merging similar values within an interval, thus reducing data redundancy. In our approach, we employ L-infinite-oriented quantization, which ensures that the error for every single pixel is bound to a specific value, known as the Maximum Absolute Difference (MAXAD). Thus, our coding scheme guarantees that the error for any pixel does not exceed a predefined MAXAD value. Moreover, the L-infinite quantization method helps preserve the semantic integrity of the scene, as the pixel value errors are controlled. As a result, the entire frame can be reconstructed with high local quality at the decoder.

For the proposed coding scheme, we have designed piecewise uniform mid-tread quantizers equipped with a deadzone. The quantization method is applied to the residual images, which contain both positive and negative values but exhibit a highly sparse (discontinuous) distribution. The piecewise uniform quantizers are adapted to the nature of the input distribution by dropping and merging redundant bins, and then the quantization indices are reassigned. Specifically, the quantization process is initialized with the following mid-tread uniform quantizers, where Δ denotes the quantization bin defined as Δ = 2.MAXAD:

(1) $$Q\left(x\right)=sign\left(x\right).\left\lfloor \frac{\left|x\right|}{\Delta }+\frac{1}{2}\right\rfloor$$

These are then updated to the nature of the distribution using a bin drop procedure described in the following steps of Algorithm 1:
**Algorithm 1** Piecewise uniform quantization procedureGiven the quantization bin Δ and number of data points *N*  Compute the number of quantization levels *M*  Compute ordered set of bin indices ${\left\{b_{k}\right\}}_{k=0}^{M}$ and bin assignments ${\left\{c_{i}\right\}}_{i=1}^{N}$  Compute bin probabilities ${\left\{p_{k}\right\}}_{k=0}^{M}$  Compute ordered set of non-empty bin indices ${\left\{b_{k}^{\prime }\right\}}_{k=0}^{M^{\prime }}$ for bins with $p_{k}>0$  Reassign bin indices ${\left\{b_{k}\right\}}_{k=1}^{M}$ to ${\left\{b_{k}^{\prime }\right\}}_{k=0}^{M^{\prime }}$  Reassign bin assignments ${\left\{c_{i}\right\}}_{i=1}^{N}$ using new indices ${\left\{b_{k}^{\prime }\right\}}_{k=0}^{M^{\prime }}$

This technique reduces the number of indices in the residual image, resulting in an increase in the compression performance. Furthermore, the foreground image, which contains critical information about the moving objects in the scene, is not quantized and remains losslessly compressed. By this technique, the essential information of the scene, particularly the moving objects, can be reconstructed without losing quality at the decoder.

In the experimental results section, we also report compression results obtained by equipping our coding scheme with uniform quantizers. In this respect it is important to point out that the method employed to encode the quantized data is slightly different between the two quantization schemes. For uniform quantizers it is sufficient to transmit the bin size to the decoder, followed by encoding of the bin indices. In contrast, for piecewise uniform quantization, the actual assignment points are also transmitted to the decoder so that the quantized values can be correctly mapped back to their original values in the residual image.

#### 3.1.3. Lossless Coding

Lossless coding represents the final stage in our architecture for completing the compression process. The algorithm chosen here is JPEG-LS because of its highly efficient way of combining predictive compression with entropy coding. Furthermore, compared to other encoders, JPEG-LS has the most straightforward implementation with low complexity on the embedded camera sensor. In our approach, encoding is conducted on a frame-by-frame basis, applying the JPEG-LS algorithm to both the residual image and the foreground image independently. Additionally, in the near-lossless coding mode, we compress the list of quantization assignment indices using the Zlib [36] library. The compressed bytes are concatenated with a header, forming a complete packet for each frame. The header contains essential metadata, including the packet ID, packet size, MAXAD value, and the compression size for each separated image. The combination of the packet ID and packet size facilitates the identification of each frame’s packet within the received stream during decoding. This packet structure facilitates efficient transmission and ensures that both the residual and foreground data can be accurately reconstructed in the decoder.

### 3.2. Decoder

The decoder block performs the inverse operation of the encoding procedure. It processes the received packets for each frame individually, extracting the residual and foreground images using the information provided in the header. For near-lossless mode, we also rearrange the quantized residual according to the assignment indices. Since the reference background is received as the first packet and stored, the background of each frame can be reconstructed by adding the residual to the reference background. In addition, the refinements applied during encoding are reversed in this stage. Finally, the foreground and background are combined to reconstruct the frame. This process ensures that the compressed data are decoded efficiently and that the frame is reconstructed accurately.

## 4. Experimental Assessment

In this section, we introduce the dataset we used for our experiments and explain the implementation of our codec in the embedded platform. Finally, we show the results of our codec compared with other algorithms capable of performing L-infinite compression.

### 4.1. Dataset

**Figure 2:**
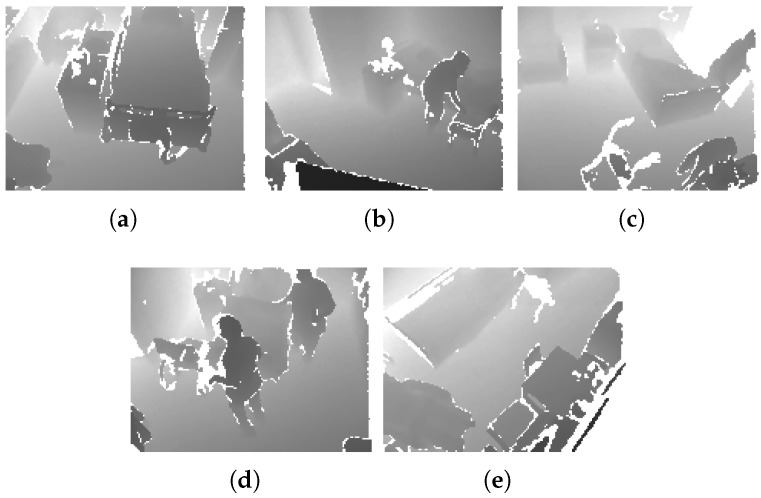
Sample depth map frames of 5 different hospital rooms: (**a**) Room 1, (**b**) Room 2, (**c**) Room 3, (**d**) Room 4, (**e**) Room 5.

**Figure 3:**
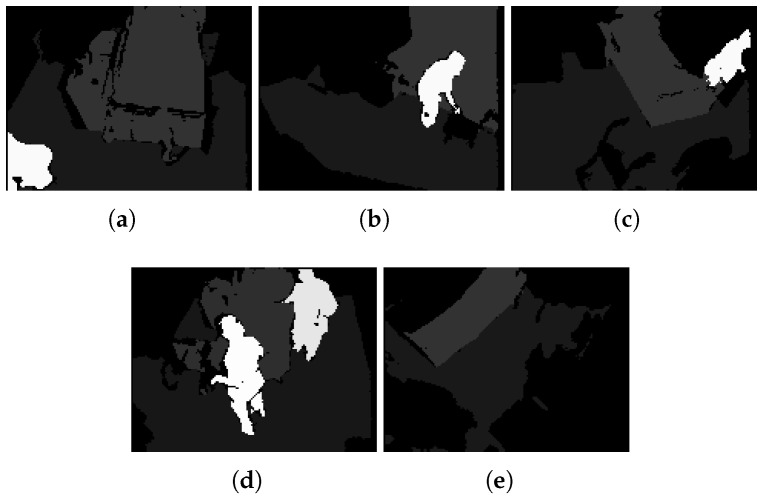
Sample segmentation map frames of 5 different hospital rooms (white color indicates moving objects in the scene): (**a**) Room 1, (**b**) Room 2, (**c**) Room 3, (**d**) Room 4, (**e**) Room 5.

The proposed codec has been evaluated using video datasets captured by the depth-camera sensor in various hospital rooms. These datasets include videos from elderly patients, nurses, beds, and other objects present in the rooms in typical monitoring setups for elderly care. Each video sequence comprises 16-bit depth maps as provided in Table 1. The depth values are represented in millimeter units. The 8-bit segmentation maps of the datasets are produced using a machine learning algorithm executed before the encoding block and are employed in order to perform foreground-background segmentation. In principle, the proposed codec can accommodate any foreground-background segmentation method. However, in our experiments, a proprietary semantic segmentation technique trained on video depth data for elderly monitoring applications has been employed. In the segmentation map, each pixel’s label corresponds to a specific object in the scene. Specifically, label 0 represents non-valid pixels (redundant pixel information of the scene), label 1 corresponds to the ground, label 2 represents the bed, if present in the scenes, and labels 10 and above denote humans, which are our moving objects in the scene. This labeling system improves the precision of the segmentation by clearly identifying static and dynamic elements within the frame. Figure 2 illustrates a sample depth frame of each dataset from 5 different hospital rooms, while Figure 3 shows the corresponding segmentation map of those sample depth frames.

### 4.2. Codec Implementation

Our proposed compression scheme features a lightweight encoder for real-world development on embedded platforms. Therefore, the encoder component capable of performing both lossless and L-infinite near-lossless compression is implemented directly in the depth-camera sensor. The depth camera is the Orbbec Persee camera sensor, which is a light-field sensor capable of capturing RGB, depth, and point cloud data from the scene. It features Ubuntu 16.04 LTS as OS with 2 GB RAM and armv7l CPU. The encoder component is implemented using the C++ language in the depth camera, while the decoder part is implemented using Python and runs on the desktop platform. The implementation ensures efficiency in real-time operations and robust processing in the camera, which we will show the runtime results later.

### 4.3. Experimental Results

The first step of the experiment is conducted in lossless mode, wherein all video frames from the datasets are encoded. The resulting bit rates (in bpp) and percentage gains are reported in Table 2. We compare our work with two other well-established lossless coding schemes, namely CALIC [37] and JPEG-LS [33].

The results in Table 2 show that our encoder, which integrates a residual-based approach with segmentation and lossless JPEG-LS coding, outperforms the standalone CALIC and JPEG-LS in terms of bit rate. The reported results prove that the additional processing has a significant effect in lowering the bit rate while losslessly preserving the input data, encoding the semantic segmentation on the input video depth, and, as shown later, offering full real-time encoding capabilities on low complexity embedded devices.

In the near-lossless mode of compression, we achieve a higher compression rate by adding the quantization block. Moreover, the use of L-infinite quantizers allows us to bind the error for every single pixel to a specific value. Here, we chose to losslessly preserve the foreground, which contains moving objects or humans, as this type of compression is necessary for accurate fall detection. Table 3, Table 4, Table 5, Table 6 and Table 7 illustrate the results obtained using our proposed L-infinite coding scheme equipped with simple uniform and piecewise uniform quantizers, respectively, while guaranteeing the depicted MAXAD values throughout the entire video sequence. Similar results are reported for the near-lossless versions of standalone CALIC and JPEG-LS that are operating at the same maximum distortion values. The results report the bit rate (in bpp) and Peak Signal-to-Noise Ratio (PSNR). For both quantization modes of the codec, we have the same PSNR because the residual images are the same.

Table 3, Table 4, Table 5, Table 6 and Table 7 show that for near-lossless L-infinite compression, the proposed codec achieves higher compression rates (lower bit rates) compared to standalone CALIC and JPEG-LS. Moreover, the results clearly show that the proposed codec outperforms both standalone CALIC and JPEG-LS in terms of PSNR. The use of L-infinite quantizers allows us to successfully lower the bit rate compared to lossless compression while maintaining a guaranteed bound on the maximum error for each pixel. Additionally, the codec design ensures that the critical part of the scene, which is the foreground, is preserved. Finally, the results demonstrate that piecewise uniform quantization outperforms uniform quantization in terms of compression performance.

**Figure 4:**
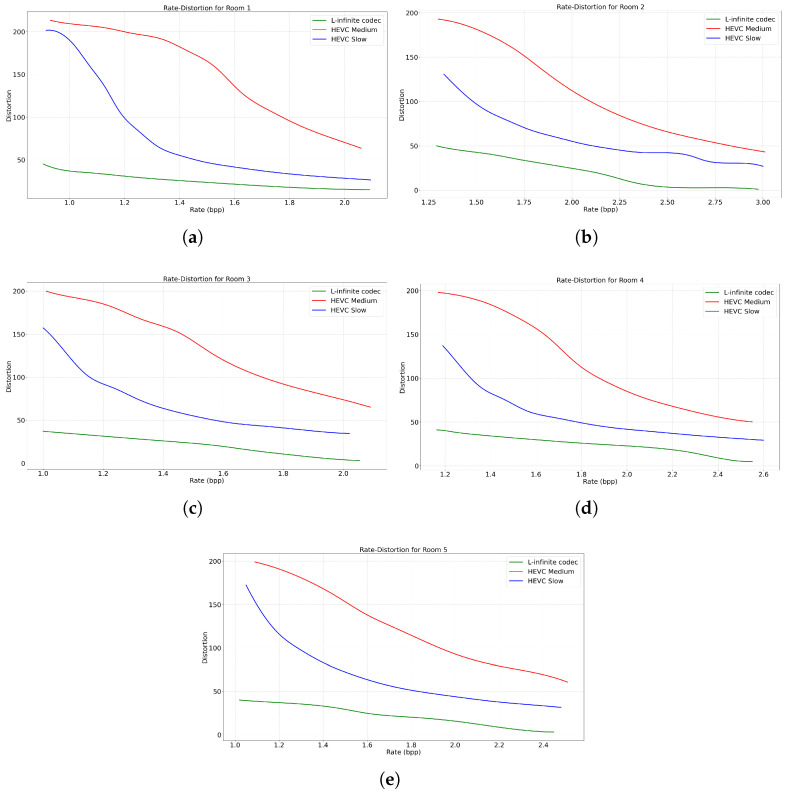
Rate-distortion curve: (**a**) Room 1, (**b**) Room 2, (**c**) Room 3, (**d**) Room 4, (**e**) Room 5.

**Figure 5:**
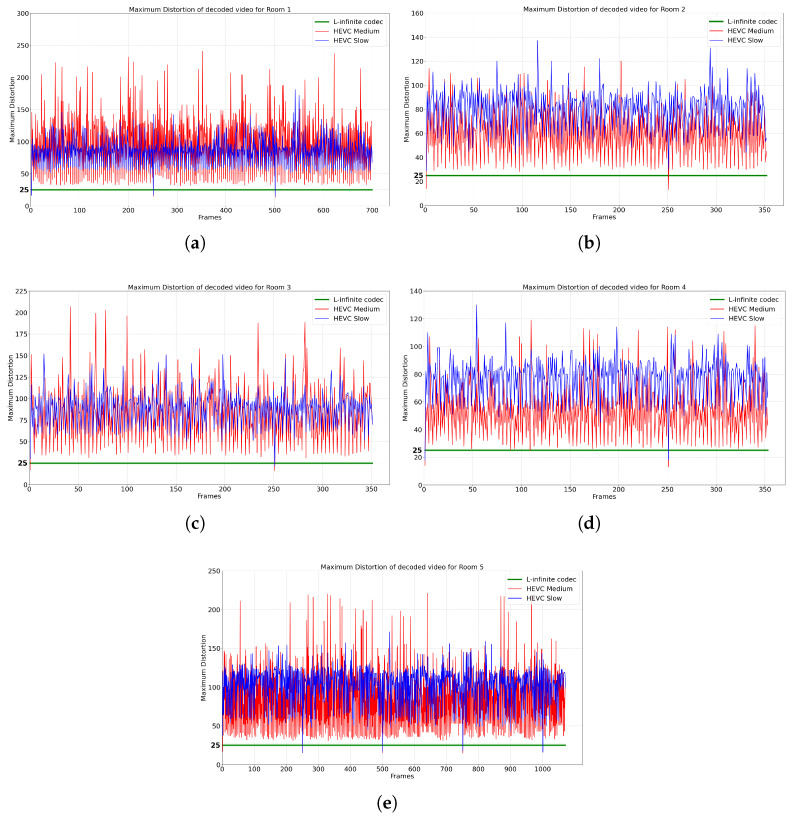
Maximum distortion of video across all frames: (**a**) Room1, (**b**) Room 2, (**c**) Room 3, (**d**) Room 4, (**e**) Room 5.

In the following, we also compare our proposed L-infinite codec with HEVC. The performance rate of HEVC varies depending on parameters such as the Constant Rate Factor (CRF) and the preset. The CRF is a parameter in the HEVC encoder within FFmpeg that determines the trade-off between compression rate and video quality. We varied the CRF value from 17 to 29, incrementing by 1, to obtain different compression rates in HEVC encoding. The preset parameter determines the encoding speed in HEVC, where a slower preset mode increases the compression rate at the cost of increasing encoding time. In the first instance, in Figure 4, we compare the Rate-Distortion (RD) curves obtained using our method, respectively HEVC operating under two encoding speed presets: medium and slow. Specifically, for the same rate, we report the maximum distortion introduced across all video frames. The choice of HEVC presets is justified as medium being the typical default value, while slow being the value that offers the highest compression efficiency. Then, in Figure 5, we focus on a specific average MAXAD value of 25 to evaluate the maximum distortion across all frames over time for an equivalent bit rate. This comparative analysis highlights the efficiency of the proposed L-infinite codec in maintaining a lower bounded local distortion while achieving competitive bit rates. This is evident from the horizontal line plotted in Figure 5, which is lower than nearly all frames processed by HEVC, regardless of its settings. As can be seen in these figures, HEVC lacks the ability to bind the distortion in the decoder, while our proposed L-infinite codec maintains the quality of the video for each frame. Moreover, at the same bit rate, on average, the proposed codec offers a lower and constant distortion bound.

The implementation of our encoder on the depth-camera sensor allows us to show-case the real-time performance of the encoder, which is compiled and optimized for fast and real-time operation in both lossless and near-lossless (L-infinite) modes. Table 8 illustrates the average runtime per frame for the encoder in all different modes for the five hospital rooms. This table confirms that our proposed codec operates with low computational complexity and is capable of compressing video data in real time. The low runtime per frame, even in near-lossless (L-infinite) mode, demonstrates the efficiency of the encoder. This efficient performance is crucial for applications like fall detection in medical settings, where both speed and accuracy are essential.

## 5. Discussion

In the first set of experiments, for all datasets, we compared the performance of our proposed method against the standalone implementations of the JPEG-LS and CALIC algorithms. In lossless mode, we observe that the proposed codec clearly outperforms both JPEG-LS and CALIC, for which we report an average improvement of 45% in bit rate over JPEG-LS and 17% over CALIC, respectively. A more detailed examination of the L-infinite mode across different datasets also shows significant gains. In Room 1, our method obtained an average improvement of 50.4% over JPEG-LS and 22.1% over CALIC for various MAXAD values. For Room 2, the improvements are 40.2% over JPEG-LS and 23.2% over CALIC; for Room 3, 48.7% over JPEG-LS and 30.5% over CALIC; for Room 4, 41.8% over JPEG-LS and 15.9% over CALIC; and for Room 5, 55% over JPEG-LS and 27.3% over CALIC. These results prove the significant impact of L-infinite quantization employed by the proposed encoder.

In the second set of experiments, it should be noted that HEVC can achieve varying compression rates depending on the encoder parameters. We compared the maximum distortion across all frames for each rate, and also we showed the rate-distortion curve for each dataset. The results show that HEVC cannot bind the distortion, while our proposed L-infinite codec consistently achieves lower and guaranteed L-infinite distortion compared to HEVC.

Furthermore, the proposed lightweight codec is shown to be highly efficient and capable of real-time operation when implemented on a depth-camera sensor, making it suitable for practical applications in medical environments. The runtime figures for the proposed codec are reported in Table 8 for all datasets in all different modes. We note that while the slower mode of HEVC provides a lower bit rate, it also costs higher encoding time, which makes it unsuitable for real-time applications on embedded devices.

## 6. Conclusions

In this paper, we have proposed a novel lightweight compression system for video depth data. Our codec is designed to operate in lossless or L-infinite near-lossless compression. In the latter case, we bound the distortion for each pixel by a specific value referred to as the Maximum Absolute Difference (MAXAD). This feature has the ability to control the coding error on each depth pixel and to preserve the semantic interpretation of the scene, which is crucial in medical applications. In both modes, we showed that the proposed codec outperforms state-of-the-art coding schemes. Furthermore, our codec performed real-time compression when implemented in an embedded platform such as a depth-camera sensor, making it practical for real-world use.

## Figures and Tables

**Table 1:** Details of depth map video sequences in our dataset.

Room No.	Frame Number	Resolution	Data Type	fps
Room 1	700	120 × 160	16-bit	3
Room 2	352	120 × 160	16-bit	3
Room 3	351	120 × 160	16-bit	3
Room 4	353	120 × 160	16-bit	3
Room 5	1071	120 × 160	16-bit	3

**Table 2:** Compression results in bit rate (in bpp) and percentage gains for lossless coding.

Room No.	JPEG-LS	CALIC	Proposed Codec	Gains over JPEG-LS	Gains over CALIC
Room 1	7.616	4.781	**4.243**	44.2%	11.2%
Room 2	7.186	4.915	**3.909**	45.6%	20.4%
Room 3	6.250	4.233	**3.170**	49.2%	25.1%
Room 4	7.177	4.566	**4.185**	41.6%	8.3%
Room 5	7.424	4.079	**3.859**	48%	5.3%

**Table 3:** Near-lossless compression results for Room 1. Rates (in bpp) and PSNR are shown, together with guaranteed maximum distortion (MAXAD) values.

MAXAD	JPEG-LS		CALIC		Proposed L-Infinite Codec		
					Uniform	Piecewise Uniform	
	Bit Rate	PSNR	Bit Rate	PSNR	Bit Rate	Bit Rate	PSNR
1	6.621	99.94	4.432	99.98	3.62	**3.029**	**120.06**
5	5.056	76.15	3.168	76.80	2.932	**2.678**	**95.22**
10	4.304	64.98	2.64	65.29	2.559	**2.456**	**82.79**
15	3.822	58.5	2.345	59.15	2.117	**2.091**	**72.92**
20	3.465	53.87	2.177	54.57	**1.669**	1.676	**67.85**
25	3.166	50.25	2.067	50.98	**1.431**	1.433	**64.79**
30	2.922	47.27	1.972	48.10	**1.264**	1.277	**62.21**

**Table 4:** Near-lossless compression results for Room 2. Rates (in bpp) and PSNR are shown, together with guaranteed maximum distortion (MAXAD) values.

MAXAD	JPEG-LS		CALIC		Proposed L-Infinite Codec		
					Uniform	Piecewise Uniform	
	Bit Rate	PSNR	Bit Rate	PSNR	Bit Rate	Bit Rate	PSNR
1	6.302	99.97	4.611	100.16	3.389	**2.975**	**119.11**
5	4.768	76.31	3.569	76.93	2.662	**2.421**	**96.01**
10	3.994	65.02	3.089	65.84	2.464	**2.297**	**84.77**
15	3.51	58.28	2.762	59.16	2.351	**2.215**	**77.34**
20	3.215	53.6	2.529	54.44	2.228	**2.12**	**73.89**
25	2.964	49.8	2.371	50.73	2.071	**1.988**	**67.58**
30	2.774	46.76	2.24	48	1.913	**1.849**	**62.85**

**Table 5:** Near-lossless compression results for Room 3. Rates (in bpp) and PSNR are shown, together with guaranteed maximum distortion (MAXAD) values.

MAXAD	JPEG-LS		CALIC		Proposed L-Infinite Codec		
					Uniform	Piecewise Uniform	
	Bit Rate	PSNR	Bit Rate	PSNR	Bit Rate	Bit Rate	PSNR
1	5.54	100.11	3.919	101.19	2.709	**2.356**	**120.55**
5	4.335	76.62	3.009	78.02	2.15	**1.959**	**96.87**
10	3.653	65.41	2.564	66.73	1.92	**1.815**	**84.47**
15	3.151	58.52	2.279	60.28	1.757	**1.692**	**78.56**
20	2.8	53.8	2.091	56	1.64	**1.587**	**73.82**
25	2.514	50.09	1.969	52.02	1.467	**1.431**	**66.83**
30	2.301	47.19	1.855	49.12	1.254	**1.248**	**61.80**

**Table 6:** Near-lossless compression results for Room 4. Rates (in bpp) and PSNR are shown, together with guaranteed maximum distortion (MAXAD) values.

MAXAD	JPEG-LS		CALIC		Proposed L-Infinite Codec		
					Uniform	Piecewise Uniform	
	Bit Rate	PSNR	Bit Rate	PSNR	Bit Rate	Bit Rate	PSNR
1	6.32	99.96	4.192	100.05	3.587	**3.07**	**119.17**
5	4.73	76.18	3.263	76.74	2.838	**2.551**	**94.59**
10	3.978	65	2.791	65.5	2.556	**2.381**	**82.71**
15	3.576	58.31	2.471	58.74	2.41	**2.286**	**78.09**
20	3.3	53.65	2.27	54.29	2.238	**2.14**	**68.75**
25	3.063	50.01	2.14	50.73	1.915	**1.852**	**62.7**
30	2.876	47.05	2.026	47.79	1.618	**1.588**	**58.93**

**Table 7:** Near-lossless compression results for Room 5. Rates (in bpp) and PSNR are shown, together with guaranteed maximum distortion (MAXAD) values.

MAXAD	JPEG-LS		CALIC		Proposed L-Infinite Codec		
					Uniform	Piecewise Uniform	
	Bit Rate	PSNR	Bit Rate	PSNR	Bit Rate	Bit Rate	PSNR
1	6.722	100.05	4.265	100.79	3.297	**2.86**	**119.3**
5	5.386	76.51	3.348	77.4	2.564	**2.317**	**95.18**
10	4.655	65.38	2.872	66.06	2.29	**2.16**	**86.31**
15	4.199	58.75	2.564	59.92	2.094	**2.02**	**74.9**
20	3.892	53.78	2.364	55	1.851	**1.811**	**71.12**
25	3.604	50.01	2.227	51.47	1.63	**1.592**	**66.22**
30	3.36	47.08	2.111	48.59	1.495	**1.483**	**61.45**

**Table 8:** Average runtime per frame of encoder, for different modes.

Room No.	Lossless (ms)	Proposed L-Infinite Codec (ms)	
		Uniform	Piecewise Uniform
Room 1	6.3	8	9.5
Room 2	6.1	7.2	9
Room 3	5.2	6.5	8
Room 4	6	7.2	8.8
Room 5	5.7	7	8.4

## Data Availability

Data are contained within the article.
